# Distinct shifts in bacteriophage diversity and abundance during various stages of Gouda-type cheese production

**DOI:** 10.1128/aem.00651-25

**Published:** 2025-08-07

**Authors:** Jun-Hyeok Yu, Gabriele Andrea Lugli, Marco Ventura, Roel van der Vaart, Arjen Nauta, Jennifer Mahony, Douwe van Sinderen

**Affiliations:** 1 School of Microbiology & APC Microbiome Ireland, University College Cork 8795, Cork, Ireland; 2 Department of Chemistry, Life Sciences and Environmental Sustainability, Laboratory of Probiogenomics, University of Parma 9370, Parma, Italy; 3 Interdepartmental Research Centre “Microbiome Research Hub”, University of Parma 9370, Parma, Italy; 4 FrieslandCampina, Amersfoort, the Netherlands; The Pennsylvania State University, University Park, Pennsylvania, USA

**Keywords:** cell wall polysaccharides, metagenomics, lactococcus, fermentation, bacteriophage

## Abstract

Bacteriophage (or phage) infection is known to disrupt milk fermentation processes involving lactic acid bacteria (LAB). This highlights the need to understand the dynamics of phage infection, particularly in fermentations involving undefined complex starter cultures, where conventional phage detection methodologies that rely on host bacteria come with limitations. Here, a metagenomic approach combined with microbiological methods was employed to assess the presence, diversity, and compositional changes in lactococcal phages within an industrial cheese production process. The virome data sets of ingredients and samples taken at various stages of the fermentation process were analyzed, revealing 12 presumptive lactococcal phage genomes belonging to either the *Skunavirus* genus or the P335 group phages. Phylogenetic analysis of the receptor-binding protein (RBP) sequence from these identified *Skunavirus* phages enabled us to predict the cell wall polysaccharide (CWPS) types of their bacterial hosts. This prediction was partially validated through host-range assays of three isolated phages, which matched phage genome contigs identified in the virome. Furthermore, alignment of their RBP-encoding gene sequences against virome data, along with real-time quantitative PCR targeting the *Skunavirus* genus, revealed a significant shift in the abundance and diversity of *Skunavirus* phages infecting different CWPS type strains throughout the production regime. Overall, our study revealed fluctuations in phage abundance and diversity during a cheese production cycle, underscoring the potential risk of inconsistent product quality due to phage proliferation and the need for effective strategies to mitigate phage contamination.

**IMPORTANCE:**

This study investigated dairy samples collected during a full fermentation cycle in a Gouda-type cheese facility to assess the prevalence, abundance, diversity, and dynamics of bacteriophages (phages) infecting starter culture components using a combination of metagenomic and microbiological assays. Also, by predicting the cell wall polysaccharide (CWPS) type of the host bacteria based on receptor-binding protein (RBP) sequences, the possible impact of bacteriophages on starter culture composition was evaluated. These findings highlight the need for tracking strategies to monitor bacteriophage dynamics in order to ensure robust and reliable fermentations.

## INTRODUCTION

Lactic acid bacteria (LAB) are a heterogeneous group of industrially important organisms, contributing to the organoleptic, nutritional, and health-associated properties of a wide variety of food products (1–3). Among these, *Lactococcus cremoris* and *Lactococcus lactis* are extensively employed as starter cultures in the dairy fermentation industry (4). Rapid acidification of milk through lactic acid production by a starter culture is key to a successful fermentation process to ensure product safety (5). Furthermore, during the ripening step, enzymes produced by these bacteria contribute to flavor development via milk protein degradation (6, 7).

Bacteriophages (or phages), especially virulent phages, pose a significant threat to industrial food production systems as they may cause bacterial cell lysis, loss of viability, and growth arrest, potentially delaying or even halting the fermentation process (8–11). Since the first description of phage infection in fermentation processes nearly a century ago by Whitehead and Cox (12), numerous studies have focused on understanding and mitigating the detrimental effects of phage infection in fermented (dairy) food production (13–17). Most lactococcal phages encountered in dairy fermentation facilities belong to one of the three groups, that is, the *Skunavirus* genus (previously termed 936 group phages), the *Ceduovirus* genus (previously termed the c2 group phages), or P335 phages (18). Among these, members of the *Skunavirus* genus are considered the most problematic lactococcal phages due to their virulence and ubiquity in dairy factory environments (10). The high prevalence of *Skunavirus* members in dairy fermentation facilities has been observed in various countries including Canada (19), Australia (20), Germany (21), and the Netherlands (22).

The host infection range of *Skunavirus* members has been shown to be dictated by the specific binding of a phage-encoded receptor binding protein (RBP) to the host cell wall polysaccharide (CWPS) moiety located on the cell surface of *L. cremoris/lactis* (23–26). Lactococcal CWPSs consist of a peptidoglycan-embedded rhamnan backbone to which a variable and surface-exposed polysaccharide pellicle (PSP) is attached. The region encoding enzymes responsible for PSP biosynthesis (e.g., glycosyltransferase) within the *cwps* operon exhibits genetic diversity between the strains, and the resulting structural diversity is the critical determinant for the specific host recognition by a given *Skunavirus* (27). Currently, four distinct *cwps* genotypes (named *cwps* A–D) and 11 C-subtypes (termed C_1-11_) have been described, and the associated structures of many of these have been elucidated (27, 28). Of note, an alternative classification of *cwps* genotypes into A to C types, each further divided into five to seven subtypes for each genotype, has been proposed (29). In any case, the undisputed variability in CWPS structures, combined with the specific binding capabilities of phage RBPs, has facilitated the classification of RBPs by amino acid sequence or protein structure analysis, in turn corresponding to the host range of a given phage (25, 26, 30).

Dairy-associated lactococcal phages originate from a variety of sources, including ingredients (such as raw milk and re-used whey) and the environment (i.e., air, work surface, etc.) (31). Several methods are currently employed to detect and enumerate phages, including cultivation/propagation-based (i.e., conventional double-layer plaque assay) and DNA-based methods (i.e., polymerase chain reaction [PCR]-based assays) (32). In addition, owing to advances in genome sequencing technologies, viral metagenome (also referred to as virome/phageome) analysis is increasingly applied to improve our understanding of phage diversity including non-propagatable phages, across various sources, such as dairy products (33, 34). Nonetheless, the large diversity of phages, which may or may not be propagatable, makes it very hard to assess fluctuations in composition and abundance of phage communities during the various stages of a given fermentation process (35, 36), thus highlighting the need for more comprehensive approaches to accurately monitor and assess both phage composition and dynamics.

In the current study, phage diversity and abundance in various dairy ingredients and samples representing different stages of a full production cycle of a Dutch cheese factory were analyzed. Viral metagenome (or virome) analysis revealed fluctuations in phage abundance and diversity across the production process. In parallel, culture-based analysis was undertaken, resulting in the isolation of three *Skunavirus* phages whose genomes correspond to various sequences identified in the analyzed viromes, each demonstrating specificity for lactococcal strains with distinct *cwps* genotypes.

## MATERIALS AND METHODS

### Sample collection

A total of 17 samples were provided by FrieslandCampina (Amersfoort, Netherlands) from a Dutch cheese production factory using a mesophilic, undefined lactococcal starter culture (referred to as “SM3” in this study). These (frozen) samples represent whey, curd, and various ingredients used in the production process (i.e., milk with whey cream, activated starter culture [thawed and pre-incubated in milk from a frozen state], two whey creams [pre- and post-sterilization], and finally, a mixture of milk, whey cream, and starter culture; Table 1).

**TABLE 1:** Description of samples obtained from the production line using the mesophilic SM3 starter culture

Sample number	Sample type	Relevant characteristics^*a*^
1	Milk with sterilized whey cream	Pasteurized
2	Starter culture	Activated culture
3	Mixture of milk, starter, and sterilized whey cream	N/A
4	Whey cream	Pre-sterilization
5	Whey cream	Post-sterilization
6	1st Whey	1st fill in cheese vat
7	2nd Whey	1st fill in buffer tank
8	3rd Whey	1st fill in drainage section
9	Curd	1st fill after pressing process
10	1st Whey	3rd fill in cheese vat
11	2nd Whey	3rd fill in buffer tank
12	3rd Whey	3rd fill in drainage section
13	Curd	3rd fill after pressing process
14	1st Whey	7th fill in cheese vat
15	2nd Whey	7th fill in buffer tank
16	3rd Whey	7th fill in drainage section
17	Curd	7th fill after pressing process

^*a*^ N/A, not applicable.

### Description of the cheese production process and sampling

**Fig 1:**
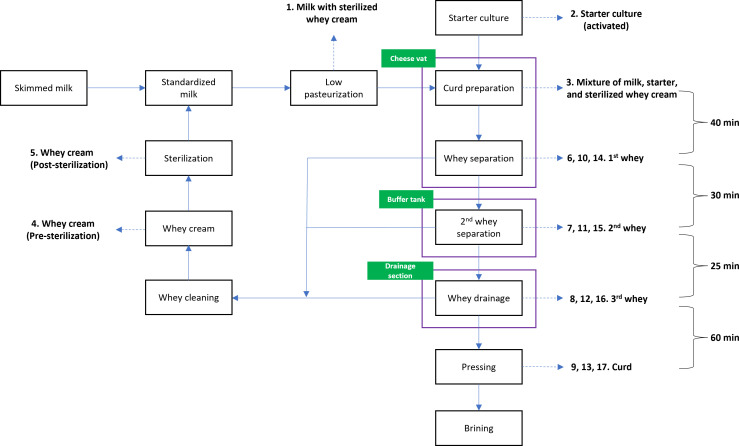
Schematic representation of cheese production process and sample collection points (as indicated by numbers) including ingredients (milk with whey cream, activated starter culture, whey cream, and a mixture of previous ingredients) and products (whey and curd). Whey and curd samples were collected at three different fill cycles (1st, 3rd, and 7th fill). For each fill, whey samples were collected three times at different time points and locations, and curd samples were collected only once after the pressing step (see text for further details).

A schematic representation of the cheese production process (across 2 days) evaluated in this study and corresponding sampling points (numbered 1–17) are presented in Fig. 1. Standardized and pasteurized skimmed milk was mixed with sterilized whey cream (sample number 1). The activated starter culture (sample number 2) and rennet were added to this mixture in the cheese vat to initiate the curdling process, after which the mixture was collected (sample number 3). During this process, whey and curd samples were collected from three different vat fills (i.e., the 1st, 3rd, and 7th fill), representing individual rounds of a production cycle from milk acidification to curd pressing. In the cheese vat, the milk was coagulated for 40 min. Afterward, the first whey was removed and collected (sample numbers 6, 10, and 14). The coagulated mixture was transferred temporarily to the buffer tank, where a second whey sample was collected (sample numbers 7, 11, and 15). After 30 min, the mixture was transferred to a drainage section, and a third whey sample was removed after 25 min (sample numbers 8, 12, and 16). Following a 60-min pressing step, the curd was collected (sample numbers 9, 13, and 17). The collected whey was recycled as whey cream (sample number 4) and sterilized at 120°C for 30 s (sample number 5). An intermediate cleaning was performed after the third fill using hot (75°C–80°C) NaOH (0.8%) for 15–17 min, in addition to rinsing the cheese vat with room temperature water between each fill.

### Sample treatment

All samples underwent a pretreatment as described previously (34). Briefly, after thawing at room temperature, the samples were diluted 10-fold with cold trisodium citrate (2% wt/vol) and homogenized in a stomacher (Lab-Blender 400, Gemini, the Netherlands) for 5 min. For solid samples, 1 g of sample was treated as an equivalent of a 1 mL liquid sample. Subsequently, the samples were centrifuged at 300 × *g* for 10 min at 4°C to remove large solid particles. The supernatant was centrifuged at 4,500 × *g* for 50 min at 4°C, and the resulting liquid fraction was subsequently filtered sequentially using 0.45- and 0.2-µM pore-sized syringe filters. The filtered solution was diluted 5-fold with cold SM buffer (50 mM Tris HCl pH 7.5, 200 mM NaCl, and 10 mM MgSO_4_) and stored at 4°C prior to subsequent assays (i.e., virome extraction and bacteriophage screening). For the enumeration of phage by qPCR and plaque assay, this dilution factor was taken into account.

### Virome DNA extraction

Total viral DNA from isolated virus-like particles (VLPs), referred to as “virome” in this study, was extracted from each processed sample as described previously with minor modifications (34). VLPs in a sample were precipitated by the addition of 10% (final conc.) polyethylene glycol (PEG8000; Sigma Aldrich, USA), followed by overnight incubation at 4°C. VLPs were then harvested by centrifugation at 15,000 × *g* for 15 min at 4°C, and the resulting pellet was resuspended in 1 mL of SM buffer. Residual host chromosomal DNA was removed by DNase (final conc. 10 U/mL) treatment for 30 min at room temperature. The reaction was terminated by adding 20 µL of 0.5 M ethylene diamine tetraacetic acid (EDTA), and the virion suspension was pretreated with proteinase K (final conc. 250 µg/mL; Fisher Scientific) for 15 min at 55°C. Phage DNA was extracted using the Phage DNA Isolation Kit (Norgen Biotek, Canada) according to the manufacturer’s instructions.

### Virome sequencing and associated sequence assembly

Virome sequencing was performed by GenProbio srl (Parma, Italy) on a NextSeq 2000 instrument (Illumina) using a paired-end 150 bp High Output sequencing kit, following library preparation with 1 ng of input DNA from each sample using the Nextera XT DNA sample preparation kit. Obtained Illumina paired-end reads were filtered using IlluQC.pl from NGSQCToolkit v2.3.3 and taxonomically classified based on the genome RefSeq and Virus RefSeq databases retrieved from NCBI. Subsequently, SPAdes v3.14 was used for *de novo* genome assembly of the classified DNA reads into contigs. The most relevant contigs (i.e., those longer than 5,000 bp) were subjected to taxonomic classification based on their sequence identity through the METAnnotatorX2 (v.2.6.5) pipeline, and open reading frames (ORFs) were predicted using Prodigal (v2.6.3) (37). For further identification, all viral contig sequences were subjected to a BLASTN (v.2.15.0) search using the NCBI BLAST tool (https://blast.ncbi.nlm.nih.gov/).

As an alternative assembly approach, following the initial assembly using SPAdes v3.14 as described above, viral metagenome assemblies were further refined using Phables v1.3.0 (38) to recover contigs corresponding to phage-related sequences. A minimum contig size cutoff of 5,000 bp was applied, whereas annotation was performed using Pharokka v1.7.3 based on the Pfam (v37.0) database (39).

### Virome data analysis

Bioinformatic analysis was performed using the MobaXterm server (https://mobaxterm.mobatek.net/). To estimate the relative abundance of each identified *Skunavirus* phage in a given sample, reads mapping was performed using CoverM v0.7.0 (https://github.com/wwood/CoverM) with the identified RBP-encoding gene sequences based on 90% alignment cutoffs with at least 97% sequence identity (40), following quality filtering of raw Illumina metagenomic data. The relative abundance of each RBP-encoding gene was normalized based on the size of each *rbp* gene (Table S1) by reads per kilobase per million mapped reads (RPKM) with 90% query coverage cut-off.

A comparative sequence analysis of the identified RBPs was performed against reference *Skunavirus* members from the NCBI database (Table S2). Amino acid (aa) sequences of the C-terminal, head domain-encompassing portion (represented by the remaining RBP sequences following removal of the N-terminal 160 amino acids) of identified RBPs were aligned using MAFFT version 7 (https://mafft.cbrc.jp/alignment/server/index.html). Phylogenetic analysis was performed using Mega (v.12) employing the neighbor-joining method, and the unrooted phylogenetic tree was generated using ITOL v7 (https://itol.embl.de/).

### Bacterial strain isolation and differentiation

LAB strains were isolated from starter SM3 using a range of nutrient media for LAB, including M17 medium (Millipore, USA) supplemented with 0.5% lactose (LM17), 2% reconstituted skim milk (RSM) supplemented with 2% glucose, and De Man, Rogosa, and Sharpe (MRS) medium (Oxoid, U.K.), as described previously (41). All cultures were incubated at 30°C. To determine the species identity of the retrieved isolates, a portion of the 16S rRNA gene of the isolates was amplified by colony PCR using a pair of specific primers (Luc *Fw*: 5′-CTTGTTACGACTTCACCC-3′ and Luc *Rv*: 5′–TGCCTAATACATGCAAGT–3′) (42). The identified *L. cremoris/lactis* strains were further subjected to plasmid profiling and *cwps* genotyping to select distinct strains (22). *L. cremoris/lactis* strains were routinely cultivated in LM17 at 30°C.

### Bacteriophage screening

Processed samples were screened for the presence of infective phages by quantitative plaque assays using the double agar method (43), employing host strains isolated in this study (Table 2). Briefly, overnight culture of each host strain was added to semi-solid (0.4% agar) LM17 supplemented with 10 mM CaCl_2_, and the mixture was plated on LM17 agar supplemented with 10 mM CaCl_2_. After overnight incubation at 30°C, individual plaques were used for phage propagations by incubation with the corresponding host strain (1% inoculum) for 3 h at 30°C in LM17 broth supplemented with 10 mM CaCl_2_. The propagated phage lysate was subjected to sequential filtration with 0.45 and 0.2 µM pore-sized syringe filters (Sarstedt Inc., Germany). Such propagated and filtered phage lysates were then used for host range analysis with the same panel of strains used in the sample screening (Table 2).

**TABLE 2:** Isolated *L. cremoris*/*lactis* strains used in this study

Strain	*cwps* genotype^*a*^	Subtype^*b*^
L1	A	N/A
L2	A	N/A
L6	A	N/A
L7	C	2
L9	A	N/A
L14	A	N/A
L19	A	N/A
L23	A	N/A
Mm2	A	N/A
Mm3	C	1
Mm4	A	N/A
Mm6	A	N/A
Mm7	A	N/A
Mm11	A	N/A
Mm14	C	2
Mm17	C	2
R15	C	2
R16	C	2
R17	C	2
SB3	U	N/A
SB31	C	4

^*a*^ U, unknown.

^*b*^ N/A, not applicable.

### Genotyping of bacteriophage isolates

To determine the lactococcal phage group/genus identity of an isolated phage, phage genomic DNA was extracted from a propagated and filtered phage lysate as described previously (34). The extracted phage DNA was then used as a template for a multiplex PCR with primer pairs specific for each of the three major lactococcal phage groups (*Skunavirus* genus, *Ceduovirus* genus, and P335 phages) (Table S3) (44). Furthermore, restriction fragment length polymorphism (RFLP) profiling of isolated phage DNA was performed with the restriction enzyme HindIII (Thermo Fisher Scientific, USA), as described previously (45). The treated DNA product was visualized on a 1% agarose gel with ultraviolet (UV) transillumination.

### Bacteriophage genome sequencing

Bacteriophage isolates exhibiting distinct host infection ranges and RFLP profiles were subjected to whole genome sequencing (performed by GenProbio srl.). A library of phage DNA fragments (250 bp) was constructed using an Illumina Nextera XT DNA Library Preparation Kit (Illumina, San Diego, CA, USA), and sequencing was performed using the Illumina MiSeq platform v3 (San Diego, CA, USA) according to the supplier’s protocol. *De novo* genome assembly and open reading frame prediction were performed as described previously (37). Predicted ORFs were annotated against the NCBI RefSeq database and INTERPRO v88.0 using DIAMOND v2.1.0 (46). The presence of tRNA genes was predicted by tRNAscan-SE v2.0 (47).

### Quantification of *Skunavirus* by qPCR

Total *Skunavirus* DNA in virome samples was quantified using real-time quantitative PCR (qPCR) with SYBR select master mix (Thermo Fisher Scientific, USA) on a LightCycler 480 system (Roche, Switzerland). The qPCR was performed employing the following conditions: 50°C for 2 min and hot start at 95°C for 2 min, followed by 40 cycles of (i) 95°C for 15 s, (ii) 55°C for 30 s, and (iii) 72°C for 30 s, using a previously described primer pair (forward: 5′-GCATTGTTCRGCTAA-AACTTT-3′ & reverse: 5′-AGCTTCGTCATACGCCTTT-AT-3′) (48). The standard curve was obtained using the reference *Skunavirus* p2 (24) (Y = −3.1777X + 38.267, R^2^ = 0.9949). Using plaque assay-derived titer of *Skunavirus* p2 as a standard, qPCR-based phage titers (Log PFU equivalents/mL or g) of samples were calculated.

### Thermal treatment assays

The thermal stability and resistance of the isolated bacteriophages were investigated using treatments that are typically applied in industry or as industry standards for phage elimination. To evaluate the thermal resistance of a given phage, 100 µL of bacteriophage lysate (10^8^–10^9^ PFU/mL) was added to 900 µL of pre-heated 10% reconstituted skimmed milk (RSM) and exposed to the following conditions: (i) room temperature × 30 min (negative control), (ii) 63°C × 30 min (low temperature, long time pasteurization), (iii) 83°C × 10 min (heat treatment employed for yogurt pasteurization), and (iv) 100°C × 1 min (highest feasible temperature to mimic whey cream sterilization), as described previously with minor modifications (35). Phage P008 was used as a reference *Skunavirus,* which has previously been shown to be temperature-sensitive (49). Following the thermal challenge, the suspension was transferred to ice, and the phage titer was determined by plaque assay. Statistical significance was evaluated using a paired one-tailed *t*-test for three independent biological experiments.

## RESULTS

### Identification of viral sequences in cheese production

#### Skunavirus-associated contigs

The extracted DNA from viral particles of each of the 17 collected samples (numbers 1–17) was sequenced, and the obtained sequence reads were assembled using MetaAnnotatorX2 (Table 3). Contig analysis revealed the presence of a total of 83 viral contigs (> 5 kb; with an average size of 11,202 bp) across the 17 samples. Of these, 62 contigs were identified as being derived from *Skunavirus* members (contigs were termed M1 through to M62; average contig size of 11,416 bp) based on BLASTN searches of the NCBI database. Also, an alternative assembly approach, using Phables (38), was performed in an attempt to recover additional lactococcal phage-related contigs. However, no further *Skunavirus*-associated contigs with unique RBP-encoding genes, beyond those already identified by MetaAnnotatorX2, were discovered (Table S4).

**TABLE 3:** Information pertaining to virome data and identified contigs from sample numbers 1–17

Sample (no.)	No. of filtered reads	No. of viral contigs (MetAnnotatorX2)	No. of Skunavirus contigs (BLASTN)	No. of P335/prophage contigs (BLASTN)
Milk with sterilized whey cream (1)	1,667	0	0	0
Starter culture (2)	80,719	2	1	1
Mixture of milk, starter, and sterilized whey cream (3)	81,361	2	0	2
Whey cream (4), pre-sterilization	60,948	6	6	0
Whey cream (5), post-sterilization	62,946	6	5	1
1st whey (6) from 1st fill	75,905	6	2	4
2nd whey (7) from 1st fill	83,113	5	2	3
3rd whey (8) from 1st fill	83,699	4	2	2
Curd (9) from 1st fill	80,948	3	1	2
1st whey (10) from 3rd fill	36,591	4	4	0
2nd whey (11) from 3rd fill	85,525	5	4	1
3rd whey (12) from 3rd fill	85,287	4	4	0
Curd (13) from 3rd fill	82,665	7	5	2
1st whey (14) from 7th fill	50,073	4	4	0
2nd whey (15) from 7th fill	80,909	7	7	0
3rd whey (16) from 7th fill	85,986	9	7	2
Curd (17) from 7th fill	83,118	9	8	1
Total	1,201,460	83	62	21

**Fig 2:**
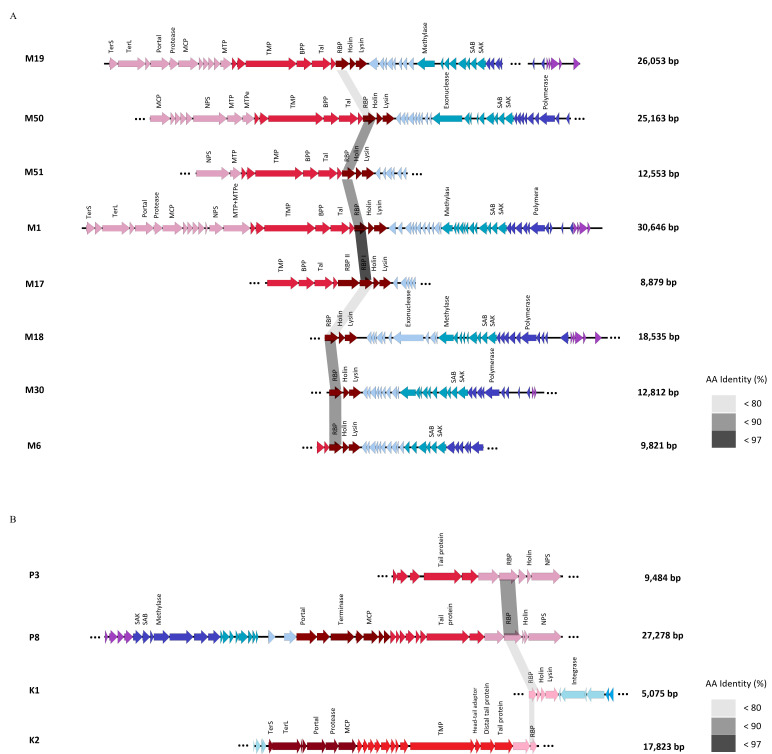
Viral contigs identified from samples 1–17. Schematic overview of eight representative *Skunavirus*-associated contigs (**A**) and P335/prophage-associated contigs (**B**). *Amino acid (AA) sequence identity was analyzed only with RBP sequences from the corresponding phages.

Among the 62 retrieved *Skunavirus*-associated contigs, 36 contigs contained single (*n* = 34) or dual (*n* = 2) RBP-encoding genes (Table S5). Among these 36 contigs, nine distinct RBP-encoding genes (< 97% identity) were identified, and the largest contig for each of these eight RBP-encoding contigs (N.B. contig M17 encompasses two *rbp* genes) was taken for further analysis (Fig. 2A). With the exception of contig M1, all eight contigs appear to represent incomplete *Skunavirus* genomes, ranging in size from 8,879 to 26,053 bp (with an average size of 11,101 bp), and encompassing 13 to 47 open reading frames. Nevertheless, these eight selected contigs appear to be consistent with a gene order also found on the reference *Skunavirus* P008 genome (49). Notably, the M17 contig contains two RBP-encoding genes, a previously described, yet rare *Skunavirus* genetic constellation (26, 50). Of these two encoded RBPs, the one with the highest level of similarity to the originally described *Skunavirus* RBP was designated as the ‘classical RBP,” designated here as RBPI. The second RBP, which contains an elongated neck region relative to RBPI and whose encoding gene is located upstream of *rbp*I, was defined as the “secondary” RBP, designated here as RBPII. The eight distinct RBP-encoding *Skunavirus*-associated contigs (i.e. M1, M6, M17, M18, M19, M30, M50, and M51) were aligned to all other *Skunavirus*-associated contigs using BLASTN to identify contigs with partially or completely overlapping sequences of near perfect identity (> 98% identity and > 20% coverage) (Fig. S1). A substantial number of the 62 retrieved contigs, including *rbp*-containing or non-containing contigs, were shown to overlap with contigs M19 and M50 (i.e., 19 out of 62, and 13 out of 62, respectively), and they were identified in all samples taken from the 3rd and 7th fills (sample numbers 10–17) (Fig. S2). Conversely, fewer contigs were shown to overlap with any of the other six representative contigs (M1, M6, M17, M18, M30, and M51). Additionally, 13 out of 62 *Skunavirus*-associated contigs did not display any sequence similarity to any of the eight representative RBP-encoding contigs, which may therefore represent non-overlapping fragments originating from the same phage genomes or the presence of additional phages (Table S5).

#### P335/prophage-associated contigs

Among the 83 retrieved viral contigs identified by MetaAnnotatorX2, 21 contigs (designated here as P1 through P21) were shown to be similar to the chromosome of lactococcal strains (which may represent prophage-encompassing regions) and to P335 phages (Table S5). Among them, two distinct RBP-encoding genes (< 90% identity) are encompassed by contigs P3 and P8, which were also shown to possess the typical gene order found in other P335-like phage genomes (Fig. 2B) (51). Furthermore, virome sequence assembly using Phables identified three additional P335/prophage-associated contigs (termed K1–K3), containing two distinct RBP-encoding genes represented by K1 and K2 (Table S4). Among the 21 P335/prophage-associated contigs identified by MetaAnnotatorX2, 10 contigs were shown to overlap with either P3 (*n* = 3) or P8 (*n* = 7), but none with K1 or K2. All contigs showing overlap with P3 or P8 were identified in samples from the 1st fill (sample numbers 6–9; Fig. S2).

### Phylogeny of the identified RBPs

**Fig 3:**
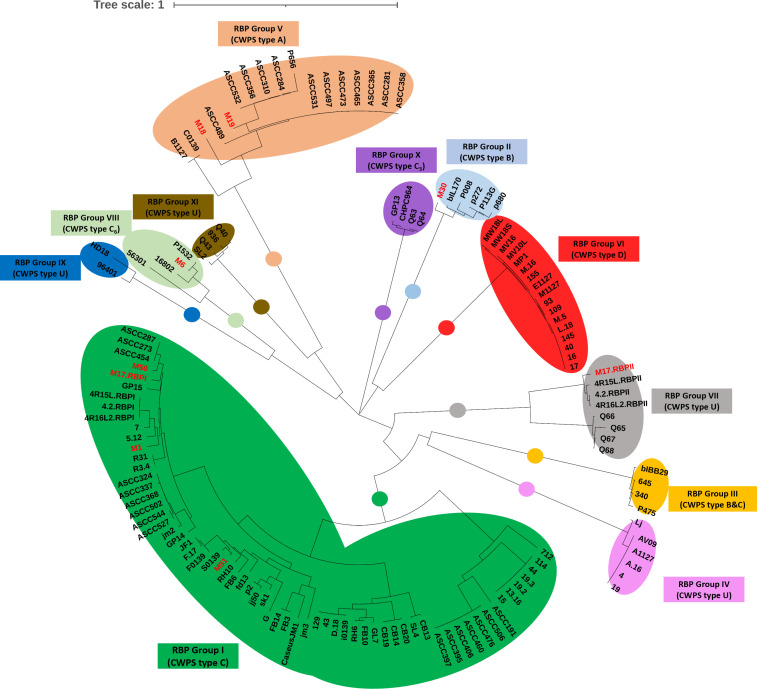
Phylogenetic tree of the C-terminal region of the RBP from *Skunavirus* phages with bootstrap values from 1,000 replicates. Red-colored text indicates RBPs identified in this study. RBP group I is marked in green, RBP group II is marked light blue, RBP group III is marked yellow, RBP group IV is marked pink, RBP group V is marked apricot, RBP group VI is marked red, RBP group VII is marked gray, RBP group VIII is marked light green, RBP group IX is marked blue, RBP group X is marked purple, and RBP group XI is marked brown. *RBPI: classical RBP, RBPII: atypical RBP.

A comparative sequence analysis of the nine *Skunavirus* RBP head domains identified in this study (Fig. 2A; NB contig M17 encodes two distinct RBPs) was performed using a large set of *Skunavirus* phage RBPs from the NCBI database (Table S2), which recently had been shown to represent 11 distinct RBP groups (Fig. 3) (30). Of the nine distinct RBPs identified in this study, four (identified in contigs M50, M1, M51, and M17-RBPI) are members of group I, one (identified in contig M30) belongs to group II, and two (identified in contigs M18 and M19) represent members of group V. Finally, the M17-RBPII and M6-RBP were shown to belong to group VII and group VIII, respectively, both of which had been newly defined in a recent study (30).

### Bacteriophage isolation and characterization

To establish the presence, number, and diversity of infective bacteriophages in samples taken at different stages of the fermentation process and from various ingredients, the collected samples were tested against selected host strains, which had been isolated from the corresponding starter culture (SM3). A total of 60 isolates were identified as *L. cremoris*/*lactis*, based on 16S rRNA gene sequencing (compositional characterization of this starter culture will be incorporated in a separate study). Subsequently, *cwps* genotyping followed by plasmid profiling (Fig. S3) was performed, allowing the selection of 21 distinct *L. cremoris/lactis* strains.

**Fig 4:**
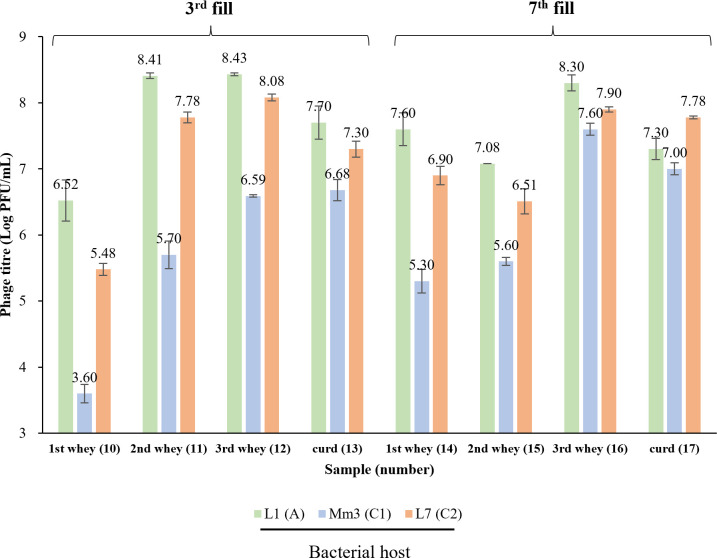
Titer of phages present in phage-positive dairy samples (numbers 10–17; Log PFU/mL) on their respective sensitive host strains. *Only representative strains for each *cwps* genotype are shown here: L1 for genotype A, Mm3 for genotype C_1_, and L7 for genotype C_2_. Phages, infecting *cwps* genotype C_4_ or unknown host bacteria, were not detected in any of the samples.

The 21 lactococcal isolates included representatives of *cwps* genotypes A, C_1_, C_2_, and C_4_ and undefined *cwps* genotypes (Table 2). Among the 17 evaluated samples, eight samples (sample numbers 10–17) were found to harbor plaque-forming phages against at least one of the strains from the panel. Although phages capable of infecting strains exhibiting *cwps* genotype A, C_1_, and C_2_ were detected, phages capable of infecting strains belonging to the C_4_ type or untyped strains were not observed (Fig. 4). Notably, strains sharing the same *cwps* genotype exhibited similar patterns and levels of phage sensitivity upon exposure to sample nos. 10–17, suggesting that they are targeted by the same phage. An increase of phage abundance was observed at the first fill whey and curd collection points against three CWPS type strains (A, C_1_, and C_2_), whereas at the second and third whey collection points, a decrease in the A- and C_2_-infecting phage abundance was observed. Based on differential plaque morphologies and distinct sample sources, a total of 18 individual phage isolates were propagated and selected for further characterization. The phage group/genus of the identified phages was assessed by multiplex PCR, revealing that all represent members of the *Skunavirus* genus (formerly the 936 group). Subsequently, to establish the number of distinct phage isolates, a host range assay was performed, demonstrating three distinct host range patterns, that is, those infecting *cwps* A (*n* = 12), C_1_ (*n* = 1), or C_2_ (*n* = 6) strains (Fig. S4A). The extracted phage genomes were further subjected to a restriction fragment length polymorphism profiling. Phages sharing the same host range profile were shown to exhibit identical RFLP profiles (Fig. S4B). Ultimately, three representative and clearly distinct phages were selected for whole genome sequencing. The generated genome sequences of the three *Skunavirus* phages were shown to correspond to the virome-based contigs M19, M50, and M51 (Table S6). The determined host range of these three *Skunavirus* members is consistent with our predicted host range as based on RBP phylogeny, that is, phage M19 infecting CWPS A type, M50 infecting CWPS type C_2_, and M51 infecting CWPS type C_1_ (Fig. 3). However, the remaining five *Skunavirus* members, as identified through virome analysis (i.e., represented by contigs M1, M17, M18, M30, and M6), could not be isolated using the available host panel. Whole genome alignment of these isolated *Skunavirus* phages against the collection of 62 *Skunavirus*-associated contigs from the virome analysis revealed seven additional contigs identical with M51, but none matching the other two (M19 and M50) (Table S5 and Fig S1).

### Tracking specific members of the *Skunavirus* population and compositional changes during cheese production

To monitor phage population abundance and compositional changes in the *Skunavirus* members during the cheese production process, we conducted a virome reads mapping combined with qPCR analysis. Viral reads were mapped using the eight *rbp* nucleotide sequences of *Skunavirus* phages identified from the virome analysis (i.e., those displayed in Fig. 2). For M17, which possesses two RBP-encoding genes, the RBPI-encoding gene (specifying a classical RBP) was used for this analysis.

**Fig 5:**
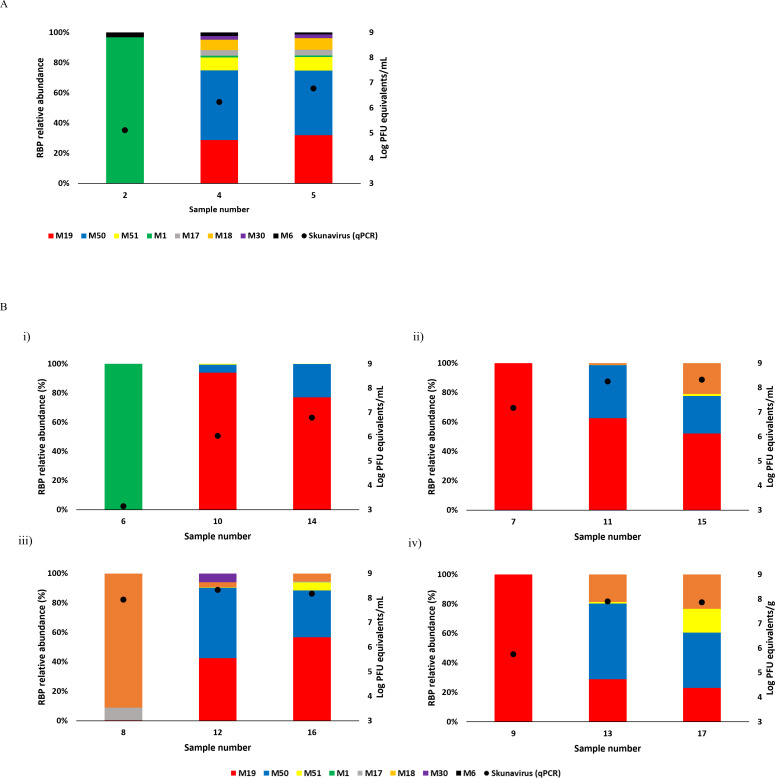
Viral compositions and abundance in ingredients samples (starter culture [sample number 2], whey cream pre-sterilization [sample number 4], and whey cream post-sterilization [sample number 5]) (**A**) and during cheese production (sample numbers 6–17) (**B**). (i) 1st whey collection (sample numbers 6, 10, and 14), (ii) 2nd whey collection (sample numbers 7, 11, and 15), (iii) 3rd whey collection (sample numbers 8, 12, and 16), and (iv) curd collection (sample numbers 9, 13, and 17).

Five ingredients associated with cheese production were analyzed, including milk with whey cream (sample number 1), activated starter culture (sample number 2), two whey cream additions pre-/post-sterilization (sample numbers 4 and 5), and a mixture of milk, whey cream, and starter culture (sample number 3) (Fig. 5A). The whey creams (sample numbers 4 and 5) appeared to contain the most diverse phage communities with higher abundance than the other ingredients (represented by sample numbers 1, 2, and 3). The whey creams showed the presence of seven and eight *rbp* sequences, respectively, which were dominated by phages corresponding to contigs M19, M50, and M51. In contrast, no reads aligned with any of the assessed *rbp* sequences were found in sample numbers 1 and 3 (data not shown). For the starter culture (sample number 2), M1 *rbp* accounted for more than 97% of the relative abundance, with a minority represented by M6 *rbp* (3%).

Changes in phage abundance were analyzed using qPCR throughout the cheese production process (sample numbers 6–17). At each sample collection point (i.e., 1st, 2nd, and 3rd whey and curd samples), an increase in the *Skunavirus* overall absolute abundance was observed in each successively sampled vat fill (Fig. 5B). These changes in absolute *Skunavirus* abundance were more significant at the 1st whey and curd collection points, showing increases of approximately 4 and 2 log units, respectively. Conversely, analysis of the 2nd and 3rd whey samples revealed a high absolute abundance of *Skunavirus* members throughout all three fills (> 7 Log PFU equivalent/mL).

Based on reads mapping, three phages (corresponding to contigs M19, M51, and M50) and contig M18 (N.B. corresponding phage was not isolated) were observed consistently throughout the entire process. In particular, phage M19 (infecting CWPS type A strain) was detected in the majority of samples (11/12) at a high relative abundance, particularly in the 2nd whey and curd at the first fill (both cases at 100% relative abundance). However, at the third fill, the relative abundance of M19 had decreased with the emergence of M50 (associated with a C_2_ type host strain) and M18, which constituted around 51% and 18% relative abundance in the curd sample, respectively. By the seventh fill, the relative abundance of M51 (infecting the C_1_ type strain) was significantly increased. Conversely, the other RBP-encoding genes (those associated with contigs M1, M17, M30, and M6) appeared sporadically with very low relative abundances in different fills and collection points. The corresponding qPCR raw data for *Skunavirus* DNA quantification are presented in Table S7.

### Thermal resistance of isolated phages

**Fig 6:**
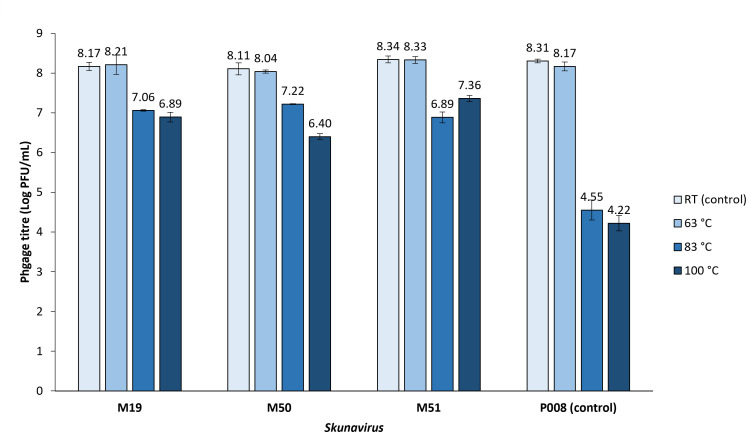
The thermal resistance profiles of isolated *Skunavirus* phages (M19, M50, and M51) in comparison to the thermally sensitive control phage P008. *Treatment conditions; (i) room temperature (RT) × 30 min (negative control), (ii) 63°C × 30 min, (iii) 83°C × 10 min, and (iv) 100°C × 1 min.

To evaluate the impact of heat treatment on phage inactivation, the isolated phages M19, M50, and M51 were subjected to dairy thermal treatments (RT, 63°C, 83°C, and 100°C) (Fig. 6). The control for this experiment was *Skunavirus* phage P008, previously shown to exhibit significant susceptibility to exposure to high temperatures (83°C and 100°C), with a reduction in phage titer (PFU/mL) of approximately 4 log units. In contrast to the control, the isolated phages showed remarkable heat resistance, exhibiting an approximately 1 log unit reduction following the treatment period.

## DISCUSSION

During milk fermentation, contamination by infective phages is a critical factor that may negatively impact the quality of the final product (52). Understanding the origin, dynamics, and diversity of phages present in the ingredients used for milk fermentation and in the fermentate during various processing steps is therefore very important. In the current study, phage presence in various samples throughout a cheese production process was investigated. Furthermore, phage abundance and compositional changes were monitored for three vat fills of a single production cycle.

In modern cheese facilities, cheese whey from a previous fermentation is often recycled to processed whey (i.e., whey cream, whey powder, etc.) and used to increase cheese yield and nutritional value of a subsequent fermentation (53–55). The use of such whey addition in cheese production brings with it a risk of phage contamination, which has prompted various attempts to eliminate phages from whey proteins (by thermal/UV treatments, membrane filtration, etc.) (56, 57). Among five ingredient samples, whey creams (sample numbers 4 and 5) were shown to contain the most diverse and highest abundance of *Skunavirus* members (Fig. 5A), compared with any other assessed ingredient; milk with sterilized whey cream (sample number 1), starter culture (sample number 2), or a mixture of all ingredients (sample number 3). Although infective phages were not detected in either pre- or post-sterilization whey cream samples using the corresponding host strains, a small quantity of *Skunavirus* members (possibly being undetectable by a culture-based method) may impose critical threats to the cheese production due to their high virulence (58). Moreover, although they may not represent the direct source of phage contamination, they could be spread indirectly through the air or by workers during the whey recycling process, as described previously (59). Also, the phage corresponding to contig M1, which accounts for 97% of relative abundance in the starter culture (sample number 1), did not appear to successfully propagate during cheese production, and the infective phage was not detected in any of the samples. However, the presence of phages in the starter culture may still implicate a significant risk of complete fermentation failure, particularly if the phage population becomes increasingly resistant to the producer’s anti-phage measures (13, 14, 35). Notably, compared with known thermoresistant lactococcal phages P680 and P1532 (60), the *Skunavirus* phages isolated in this study were shown to exhibit a similar level of heat resistance, remaining detectable after heat treatment at 100°C for 1 min with less than 2 log (PFU/mL) reduction, as illustrated in Fig. 6. These results highlight the need for more effective anti-phage strategies and stricter contamination control measures during ingredient processing to prevent *Skunavirus* contamination and subsequent propagation and ensure the integrity of cheese production.

Phylogenetic comparison of the RBP head domains enabled the prediction of CWPS types of the presumed host strains of the eight identified *Skunavirus* phages (Fig. 3). This prediction was partially validated experimentally through host range analysis of subsequently isolated phages (i.e., in the case of M19, M50, and M51), presenting infection specificity to CWPS type A, C_2_, or C_1_ host bacteria, respectively. This prediction demonstrated that most RBPs identified in this study were clustered in group I (*n* = 4) or group V (*n* = 2), corresponding to CWPS type C and A, respectively. The following reads-mapping of *rbp* sequences to the viromes of whey and curd samples also revealed the dominant presence of C and A type-infecting phages (Fig. 5B), and this dominance was further confirmed by phage isolation. For example, in the curd sample from the 1st vat fill, phage M19, which infects CWPS type A hosts, was dominant, showing 100% relative abundance. These results may reflect the possible dominance of lactococcal host strains with CWPS types C and A in this undefined starter culture, which may also explain the CWPS type distribution of our isolated host panel (Table 2). However, by the time the 3rd fill process progressed, the relative abundances of phages M50 and M18 (infecting CWPS types C_2_ and A, respectively) had increased significantly, correlating with a rise in the absolute abundance of *Skunavirus* as detected by qPCR. Finally, at the 7th fill, with the emergence of phage M51 which infects CWPS type C_1_, the viral composition of curd was shown to be more variable, compared with the 1st fill curd. These changes in phage composition may reflect significant shifts in microbial composition, potentially driven by the suppression of dominant strains through phage predation, according to the “kill-the-winner” hypothesis (61, 62). Given the potential functions of *Lactococcus* in cheese ripening (7, 63), such compositional changes in the starter culture within the curd may cause inconsistencies in product quality. However, the behavior of phages during cheese production, as observed in this study, may vary depending on seasonal or other environmental conditions, highlighting the importance of further studies. Furthermore, incorporating the analyses of bacterial communities by tracking fluctuation in the host CWPS composition as well as their abundance through metagenomics and qPCR would allow further in-depth insights into how phage infection precisely impacts the starter community composition during the fermentation process.

Phage enumeration by plaque assays showed that phage abundances are consistent with those obtained by PCR analyses for samples from the 3rd and 7th fills (Fig. 4 and 5B). However, no infectious phages were detected using plaque assay testing employing the host panel for the 1st fill samples, despite high titers of *Skunavirus* being observed by qPCR analysis (except for the 1st whey). Given that samples taken from the 2nd whey and curd samples from the 1st fill were shown to be dominated by M19, which infects CWPS type A host strains, at titers of around 7 and 6 PFU equivalents/mL or g, these results suggest that phages detected by qPCR in the 1st fill samples may have represented inactivated phages, possibly present as non-infectious particles (rather than as exogenous DNA, since the samples were treated with DNase). These inactivated phages may have arisen as a result of various practices applied in this dairy fermentation facility. For instance, sterilized whey from previous fermentations is added to the buffer tank, where the coagulated curd, transferred from the cheese vat, is held (Fig. 1) to achieve the desired density and uniform characteristics of the curd. This practice may also account for the drastic variations in *rbp* relative abundance observed in the 1st fill samples. Consequently, these results underscore the challenges in analyzing dairy samples from industrial settings by qPCR and the need to account for such industry-specific practices by incorporating alternative methods to detect infective phages.

In summary, we tracked phage composition, dynamics, and diversity throughout the cheese production process. As the process progressed, the viral composition became more diverse and their abundance increased, which may impact the microbial composition. Although we could not pinpoint the exact origin of phage contamination, whey creams were demonstrated to have the most diverse viral composition. This underscores the significant risk associated with using processed whey in cheese production. Therefore, it is crucial to continuously monitor and trace phage propagation to prevent ongoing phage infections in the industry environment.

## Data Availability

The genome sequences of the isolated phages have been deposited in GenBank under accession numbers PQ675600 (M19), PQ675601 (M50), and PQ675602 (M51) (see Table S6). The raw metagenomic reads of all samples (sample numbers 1 to 17) were deposited in GenBank under BioProject accession number PRJNA1210180.

## SUPPLEMENTAL MATERIAL

Supplemental material aem.00651-25-s0001.pdf Tables S1 to S7 and Figures S1 to S4.

ASM does not own the copyrights to Supplemental Material that may be linked to, or accessed through, an article. The authors have granted ASM a non-exclusive, world-wide license to publish the Supplemental Material files. Please contact the corresponding author directly for reuse.
