# Supplementary material for: Distinct shifts in bacteriophage diversity and abundance during various stages of Gouda-type cheese production
Source: Appl Environ Microbiol. 2025 Aug 7;91(9):e00651-25. doi: 10.1128/aem.00651-25 (PMC12442387; doi:10.1128/aem.00651-25)
Supplement: Supplemental material — Tables S1 to S7 and Figures S1 to S4. [file aem.00651-25-s0001.pdf]

Supplementary information (Table)

Table S1. The size of *rbp* nucleotide sequence used for reads mapping.

Phage	Size of *rbp* (bp)
M1	795
M17*	795
M18	819
M19	819
M30	804
M50	795
M51	795
M6	789

*The RBPI of M17 was used for reads mapping.

Table S2. Reference *Skunavirus* phages used for phylogenetic analysis.

Phage	Accession No.	Protein ID of RBP
4.2	NC_049362	YP_009874995 (RBPI) & YP_009874996 (RBPII)
5.12	NC_049357	YP_009874717
7	NC_021855	YP_008318222
13.16	KP793116	ALM63797
15	NC_049352	YP_009874453
16	KP793135	ALM64809
17	KP793114	ALM63690
19.2	KP793111	ALM64863
19.3	NC_049356	YP_009874664
40	KP793127	ALM64364
43	KP793110	ALM63524
44	NC_049363	YP_009875054
93	KM091443	AIK68612
109	NC_049360	YP_009874884
114	KP793115	ALM63745
129	KP793112	ALM63578
145	NC_049353	YP_009874509
155	NC_049366	YP_009875223
712	NC_008370	YP_764279
16802	NC_049398	YP_009877891
56301	NC_049405	YP_009878275
96401	NC_049413	YP_009878689
4R15L	KX379667	ANY28867 (RBPI) & ANY28868 (RBPII)
4R16L2	KX379668	ANY28809 (RBPI) & ANY28810 (RBPII)
ASCC191	NC_017688	YP_006201103
ASCC273	NC_017695	YP_006201552
ASCC281	NC_017702	YP_006201952
ASCC284	JQ740790	AFE86929
ASCC287	JQ740791	AFE86986
ASCC310	JQ740792	AFE87044
ASCC324	JQ740793	AFE87099
ASCC337	JQ740794	AFE87157
ASCC356	JQ740795	AFE87215
ASCC358	JQ740796	AFE87269
ASCC365	JQ740797	AFE87326
ASCC368	JQ740798	AFE87383
ASCC395	JQ740799	AFE87441
ASCC397	JQ740800	AFE87499
ASCC406	JQ740801	AFE87558
ASCC454	JQ740802	AFE87617
ASCC460	JQ740803	AFE87675
ASCC465	NC_017698	YP_006201722

Table S2. *Continued.*

Phage	Accession No.	Protein ID of RBP
ASCC473	JQ740805	AFE86644
ASCC476	JQ740806	AFE87734
ASCC489	JQ740807	AFE86701
ASCC497	JQ740808	AFE87793
ASCC502	JQ740809	AFE87849
ASCC506	JQ740810	AFE87907
ASCC527	JQ740811	AFE87965
ASCC531	JQ740812	AFE88023
ASCC532	NC_017696	YP_006201610
ASCC544	JQ740814	AFE88135
B1127	NC_049355	YP_009874612
bIL170	NC_001909	NP_047133
C0139	KP793109	ALM63472
CaseusJM1	NC_049351	YP_009874401
CB13	NC_013155	YP_003127307
CB14	NC_013152	YP_003127153
CB19	NC_013153	YP_003127205
CB20	NC_013154	YP_003127256
CHPC964	NC_049487	YP_009885839
D.18	KP793107	ALM63365
E1127	NC_049367	YP_009875277
F.17	KP793113	ALM63633
F0139	NC_049358	YP_009874770
FB10	MW041640	QPL22593
FB14	MW032477	QPL22648
FB3	MW041632	QPL22141
FB6	MW041633	QPL22197
fd13	NC_049347	YP_009874152
G	KP793117	ALM63852
GL7	MW041638	QPL22477
GP14	MW041634	QPL22256
GP15	MW041635	QPL22311
HD18[a]	-	AAT81490
i0139	NC_049375	YP_009875766
JF1	NC_049365	YP_009875165
jj50	NC_008371	YP_764332
jm2	NC_021860	YP_008320102
jm3	NC_021854	YP_008318169
L.18	NC_049359	YP_009874828
M.16	KP793128	ALM64420
M.5	KP793126	ALM64305
M1127	NC_049368	YP_009875339

Table S2. *Continued.*

Phage	Accession No.	Protein ID of RBP
MP1	NC_049434	YP_009880370
MV10L	NC_049447	YP_009882394
MV16	MK301441	QBQ81953
MW18L	KX379673	ANY28532
MW18S	KX346250	AOQ30281
P008	NC_008363	YP_762531
P113G	KC182548	AGI10973
P1532	NC_049474	YP_009884903
p2	NC_042024	YP_009613498
p272	NC_049348	YP_009874204
P656	NC_049475	YP_009884948
P680	NC_021852	YP_008318062
Q40[a]	-	AAT81506
Q43[a]	-	AAT81510
Q63[a]	-	AAT81487
Q64[a]	-	AAT81486
Q65[a]	-	AAT81520
Q66[a]	-	AAT81519
Q67[a]	-	AAT81518
Q68[a]	-	AAT81517
R3.4	KY554760	ARM65521
R31	NC_049381	YP_009876356
RH10	MW041636	QPL22367
RH6	MW041639	QPL22538
S0139	NC_049370	YP_009875444
sk1	NC_001835	NP_044964
SL2[a]	-	AAT81493
SL4	NC_028900	YP_009208054

[a]Indicates phage strains for which the corresponding whole genome sequences are not present in NCBI database.

Table S3. Primers used lactococcal phage genotyping.

Target locus		Primer	Sequence (5' to 3')	Amplicon size (bp)	Reference
Lactococcal phage group	*Skunavirus*	936A	TCAATGGAAGACCAAGCGGA	179	Labrie et al., 2000
		936B	GTAGGAGACCAACCCAAGCC		
	Ceduovirus	c2A	CAGGTGTAAAAGTTCGAGAACT	474	
		c2B	CAGATAATGCACCTGAATCA		
	P335 phage	P335A	GAAGCTAGGCGAATCAGTAA	682	
		P335B	GATTGCCATTTGCGCTCTGA		

Table S4. Identified viral contigs by Phables.

Sample	No. viral contigs	No. contig with *rbp* gene	No. contig with novel rbp gene
1	0	0	0
2	272	1	0
3	171	0	0
4	22	4	0
5	143	3	0
6	135	2	1 (K1)
7	147	2	1 (K2)
8	72	4	1 (K3)
9	64	1	0
10	9	2	0
11	8	4	0
12	22	5	0
13	62	4	0
14	10	2	0
15	16	4	0
16	14	5	0
17	11	4	0

Table S5. Viral contigs identified in this study.

Contig	Sample	Size (bp)	Coverage	No. *rbp*	Contig group
***Skunavirus*-associated**					
M1	2	30646	66	1	M1
M2	4	5038	11	1	M17
M3	4	7833	216	0	M50
M4	4	9008	159	1	M19
M5	4	17942	145	1	M50
M6	4	9821	35	1	M6
M7	4	6698	57	1	M51
M8	5	9810	280	0	M19
M9	5	6656	479	0	M19
M10	5	6097	277	1	M19
M11	5	13604	384	1	M50
M12	5	7017	83	0	M51[a]
M13	6	7408	9	0	M1
M14	6	19684	8	1	M1
M15	7	18001	23709	1	M19
M16	7	5151	18742	0	N/A
M17	8	8879	661	2	M17
M18	8	18535	6587	1	M18
M19	9	26053	234	1	M19
M20	10	20755	1046	1	M19
M21	10	15521	70	1	M50
M22	10	5699	6	1	M51
M23	10	5006	767	0	N/A
M24	11	15322	24175	1	M19
M25	11	5980	30229	0	M19
M26	11	22026	20563	1	M50
M27	11	5695	32	0	M51[a]
M28	12	11053	7644	1	M19
M29	12	15577	10048	1	M50
M30	12	12812	3016	1	M30
M31	12	6173	49	0	M51[a]
M32	13	11578	5160	1	M19
M33	13	5809	3822	0	M19
M34	13	21902	11479	1	M50
M35	13	13836	3582	1	M18
M36	13	6173	186	0	M51[a]
M37	14	5279	316	0	M50
M38	14	7920	1496	0	M19
M39	14	18723	859	1	M19
M40	14	16211	333	1	M50
M41	15	7369	4107	0	M50

Table S5. *Continued.*

Contig	Sample	Size (bp)	Coverage	No. *rbp*	Contig group
		***Skunavirus*-associated**			
M42	15	11578	8883	1	M19
M43	15	7222	7241	0	M19
M44	15	15071	4597	1	M50
M45	15	10288	4213	0	M18
M46	15	7886	3940	0	N/A
M47	15	5490	262	0	M51[a]
M48	16	7682	91	2	M17
M49	16	14042	6706	1	M19
M50	16	25163	5200	1	M50
M51	16	12553	1702	1	M51
M52	16	6907	2013	0	N/A
M53	16	6760	762	0	M51[a]
M54	16	5322	414	0	N/A
M55	17	5198	7187	0	N/A
M56	17	13045	3664	1	M19
M57	17	5887	2546	0	M19
M58	17	17838	6385	1	M50
M59	17	12067	1983	0	M51[a]
M60	17	11586	3474	1	M18
M61	17	8114	3007	0	M18
M62	17	7800	2898	1	M51
		**P335/prophage-associated**			
P1	2	5089	47	0	N/A
P2	3	7006	24	0	N/A
P3	3	9484	50	1	P3
P4	5	17927	12	0	N/A
P5	6	12502	538	0	N/A
P6	6	7410	491	0	N/A
P7	6	9459	349	0	N/A
P8	6	27278	930	1	P8
P9	7	18490	88	1	P8
P10	7	6051	8	0	P8
P11	7	5033	61	0	N/A
P12	8	20432	252	1	P8
P13	8	18008	213	0	N/A
P14	9	7655	346	1	P8
P15	9	6824	270	0	P3
P16	11	5153	100	0	N/A
P17	13	6823	26	0	P3
P18	13	7673	46	0	P8
P19	16	5177	15	0	N/A

Table S5. *Continued.*

Contig	Sample	Size (bp)	Coverage	No. *rbp*	Contig group
**P335/prophage-associated**					
P20	16	10805	39	0	N/A
P21	17	7674	43	0	P8

[a]Contigs identified by the alignment with whole genome sequence of isolated phage. *N/A: not applicable.

Table S6. Genetic information of isolated *Skunavirus* phages.

Phage	Genome size (bp)	No. gene	Accession no.
M19	28027	49	PQ675600
M50	31359	53	PQ675601
M51	30732	55	PQ675602

Table S7. Raw qPCR data for *Skunavirus* DNA quantification.

Sample (number)	Cp value		
	1st repeat	2nd repeat	3rd repeat
Milk with sterilized whey cream (1)	23.34	23.3	23.61
Starter culture (2)	21.92	22.09	22.1
Mixture of milk, starter, and sterilized whey cream (3)	26.97	26.99	27.04
Whey cream pre-sterilization (4)	18.28	18.5	18.58
Whey cream post-sterilization (5)	16.9	16.61	16.63
1st Whey from 1st fill (6)	28.53	28.06	28.29
2nd Whey from 1st fill (7)	15.56	15.45	15.44
3rd Whey from 1st fill (8)	13.27	13	12.94
Curd from 1st fill (9)	20.29	19.78	19.91
1st Whey from 3rd fill (10)	19.07	18.98	19.19
2nd Whey from 3rd fill (11)	12.05	12.04	12.08
3rd Whey from 3rd fill (12)	11.81	11.85	11.78
Curd from 3rd fill (13)	13.66	12.87	12.96
1st Whey from 7th fill (14)	16.65	16.74	16.79
2nd Whey from 7th fill (15)	11.79	11.87	11.82
3rd Whey from 7th fill (16)	12.12	12.21	12.54
Curd from 7th fill (17)	13.18	13.24	13.44

Supplementary information (Figure)

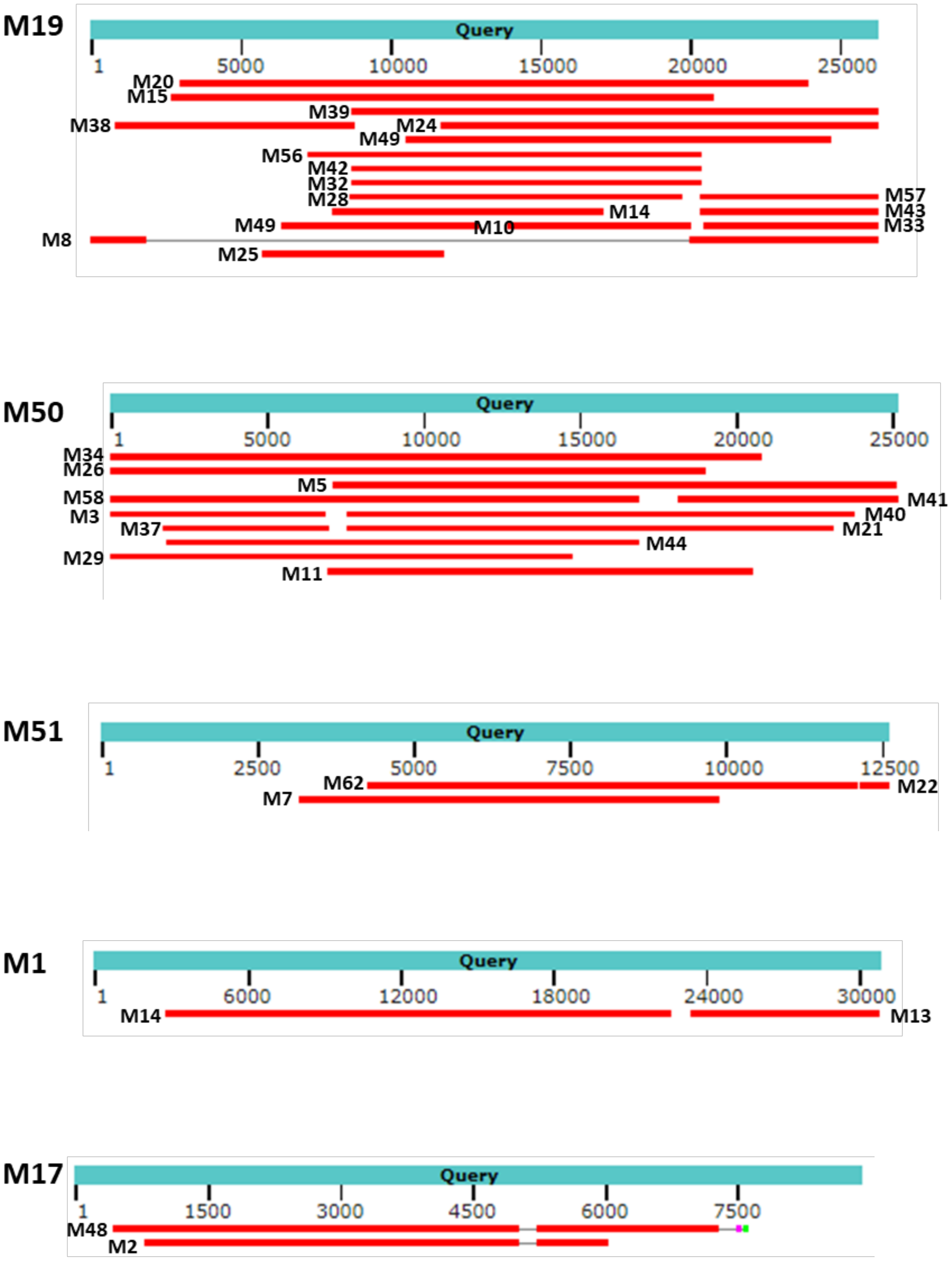

Figure S1. Alignment of eight representative contigs against other *Skunavirus*-associated contigs. Contig M30 and M6 were excluded, as there was no contig showing similarities. [a]Whole genome of phage M51.

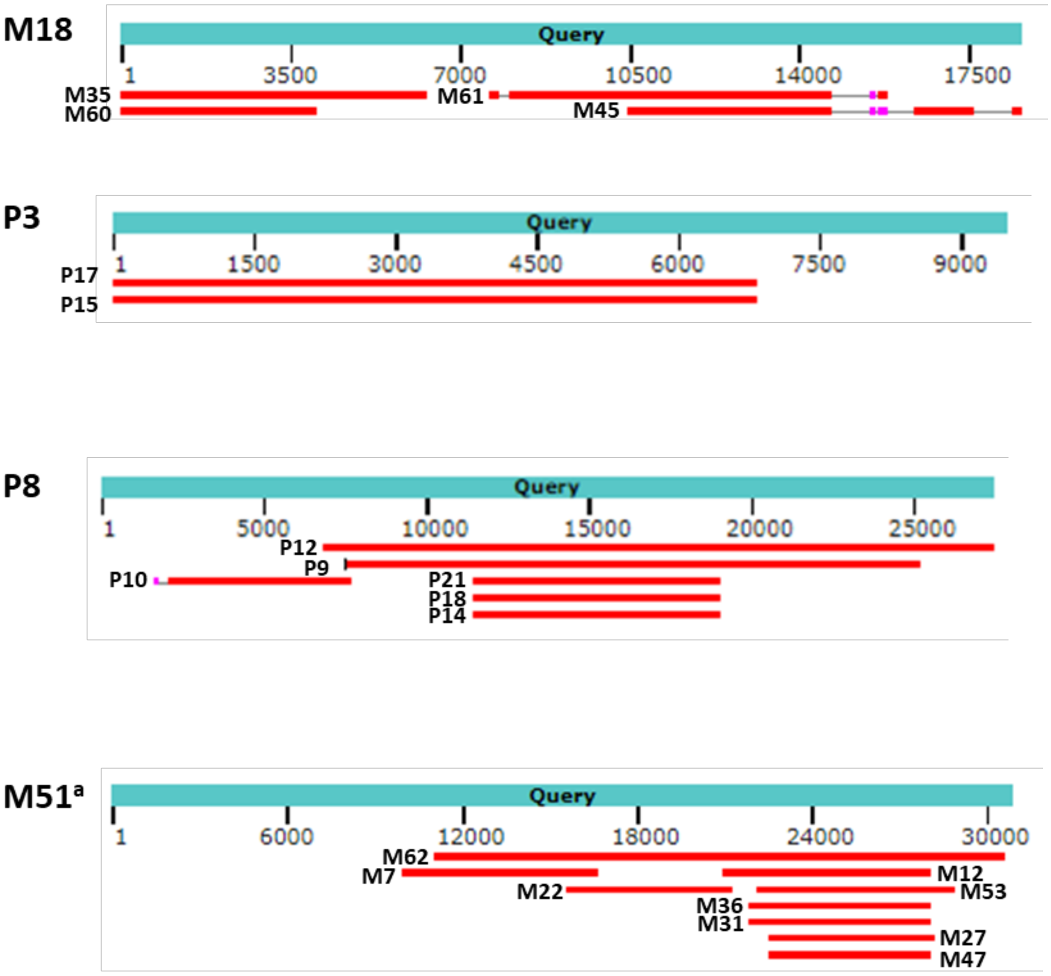

Figure S1. *Continued.*

Viral contig		Sample (number)																	Total contig
		Milk with sterilized whey cream (1)	Starter culture (2)	Mixture of milk, starter, and sterilized whey cream (3)	Whey cream before sterilization (4)	Whey cream after sterilization (5)	1st whey (6)	2nd whey (7)	3rd whey (8)	Curd (9)	1st whey (10)	2nd whey (11)	3rd whey (12)	Curd (13)	1st whey (14)	2nd whey (15)	3rd whey (16)	Curd (17)	
Skunavirus-associated	M19				1	3		1		1	1	2	1	2	2	2	1	2	19
	M50				2	1					1	1	1	1	2	2	1	1	13
	M51				1						1						1	1	4
	M1		1				2												3
	M17				1				1								1		3
	M18								1					1		1		2	5
	M30												1						1
	M6				1														1
	Unknown					1		1			1	1	1	1		2	3	2	13
P335/ prophage-associated	P3			1						1				1					3
	P8						1	2	1	1				1				1	7
	K1																		0
	K2																		0
	Unknown		1	1		1	3	1	1			1					2		11
Total contig		0	2	2	6	6	6	5	4	3	4	5	4	7	4	7	9	9	83

Figure S2. Presence or absence of contigs presenting similarities with representative contigs. *Grey-coloured box indicates presence of contig. [a]Indicates a number of contig overlapped with representative viral contigs.

A-1

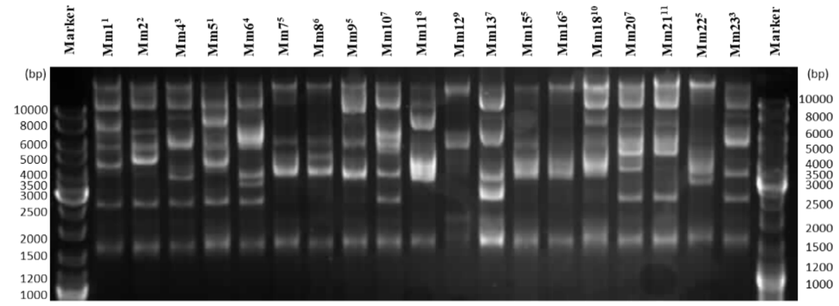

A-2

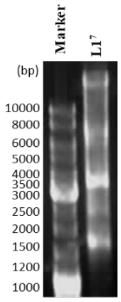

A-3

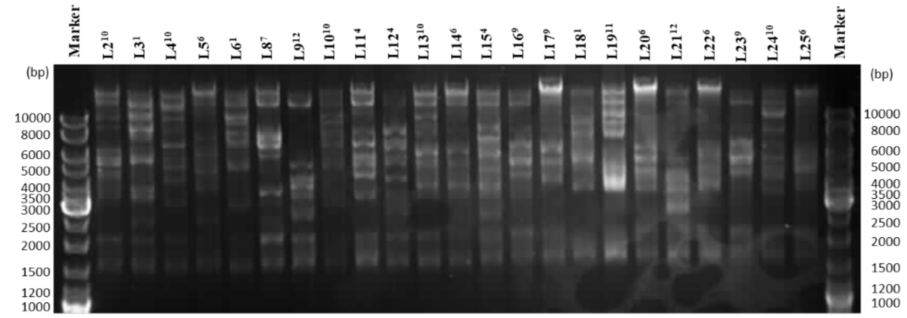

B

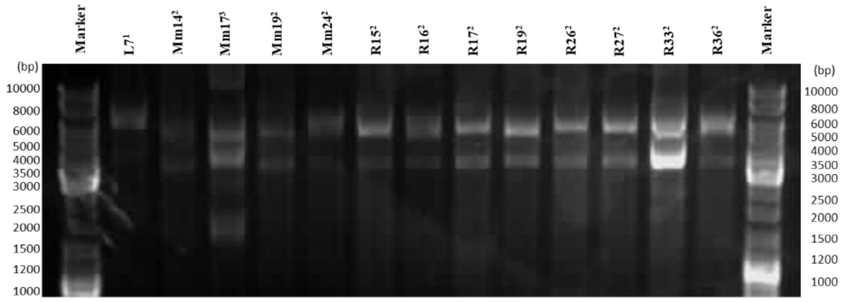

C

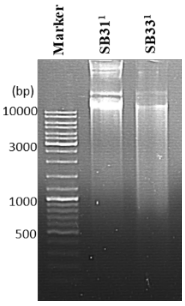

Figure S3. Plasmid profiles of *L. cremoris/lactis* isolates presenting *cwps* genotype A (Panel A-1-3), C_2 (Panel B), and C_4 (Panel C) types. ^{n}: indicates the strains presenting similar plasmid patterns (numbered 1-12). **cwps* genotype C_1 strain was not included in this figure, as only one strain was isolated.

A

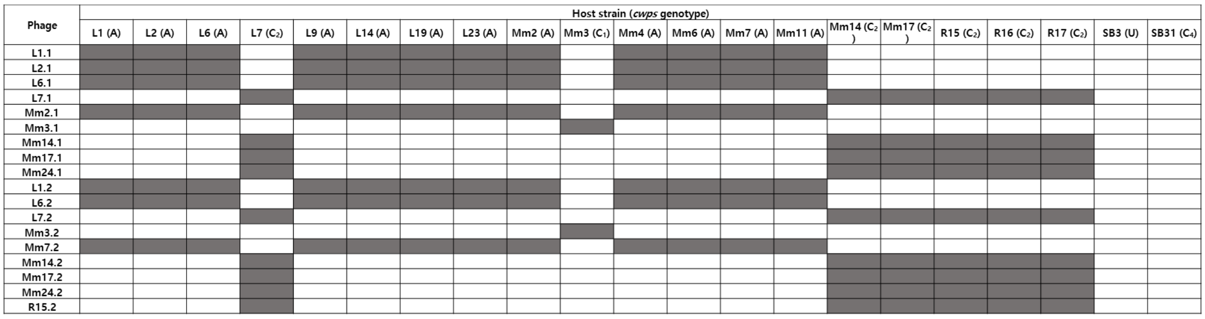

B

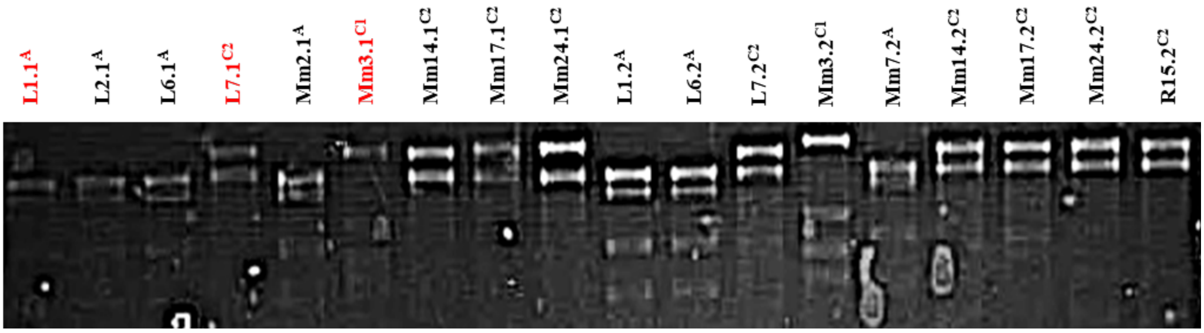

Figure S4. Host range of *L. lactis/cremoris* phage isolates (A). *Grey-filled boxes indicate strains that are susceptible to the respective phages. Restriction fragment length polymorphism (RFLP) with genomic DNA using HindIII (B). ^{X}; indicates *cwps* genotype of host bacteria. *Red-coloured label indicates phages that were subjected to whole-genome sequencing. Phage L1.1 was identified as M19, L7.1 as M50, and Mm3.1 as M51.
